# Gothenburg direct observation tool for assessing person-centred care (GDOT-PCC): evaluation of inter-rater reliability

**DOI:** 10.1136/bmjopen-2024-096576

**Published:** 2025-04-17

**Authors:** Nina Ekman, Andreas Fors, Philip Moons, Charles Taft

**Affiliations:** 1 Institute of Health and Care Sciences, Sahlgrenska Academy, University of Gothenburg, Goteborg, Sweden; 2 University of Gothenburg Centre for Person-Centred Care (GPCC), Sahlgrenska Academy, University of Gothenburg, Gothenburg, Sweden; 3 Department of Public Health and Primary Care, Faculty of Medicine, KU Leuven, Leuven, Belgium; 4 Region Västra Götaland, Research, Education, Development and Innovation, Primary Health Care, Gothenborg, Sweden; 5 Department of Pediatrics and Child Health, University of Cape Town, Cape Town, South Africa

**Keywords:** Person-Centered Care, Cross-Sectional Studies, Health, Health Education

## Abstract

**Abstract:**

**Objective:**

To assess the inter-rater reliability of the Gothenburg direct observation tool-person-centred care in assessing healthcare professionals’ competency in delivering person-centred care (PCC).

**Design:**

Observational, fully-crossed inter-rater reliability study.

**Setting:**

The study was conducted between October and December 2022 at the participants’ homes or offices.

**Participants and methods:**

Six health professionals individually rated 10 video-recorded, simulated consultations against the 53-item, 15-domain tool covering four major areas: PCC activities, clinician manner, clinician skills and PCC goals. Cronbach’s α was used to assess internal consistency. Intraclass correlations (ICC) and 95% CI were computed for the domains.

**Results:**

Two domains (planning and documentation and documentation) were excluded from analyses due to insufficient evaluable data. Cronbach’s α was acceptable (>0.70) for all evaluated domains. ICC values were high (ICC ≥0.75) for 11 of the 13 domains; however, CIs were generally wide and the lower bounds fell within the good range (ICC=0.60–0.74) for six domains and fair agreement (ICC=0.40–0.59) for the remaining six. The ICC for the domain patient perspective was non-informative due to its wide CIs (ICC=0.74 (0.39–0.92)).

**Conclusion:**

ICC estimates for most domains were comparable to or exceeded those reported for similar direct observation tools for assessing PCC, suggesting that they may reliably be used in, for example, education and quality improvement applications. Reliability for the domains planning and documentation and documentation needs to be assessed in studies sampling more documentation behaviours. Reliability for the patient perspective domain may owe to methodological issues and should be reassessed in larger, better-designed studies.

**STRENGTHS AND LIMITATIONS OF THIS STUDY**

- A strength of the study is that sample size requirements were estimated using a recognised method designed specifically for use in intraclass correlations studies and which allows for trade-offs between a number of raters and subjects to achieve adequate power.
- Another strength is that the sampled consultations covered a range of topics, settings and professional categories; however, the sample included too few examples of documentation behaviours to assess reliabilities associated with the domains planning and documentation and documentation.
- A limitation is that all participants (raters) were women, hence our results may not be generalisable to men.
- Another limitation is that ratings were performed using video-recorded consultations, hence our results may not apply to real-time consultations.

## Introduction

 Direct observation of trainees performing patient care and clinical activities is a common assessment strategy in health education and training and is now mandated by several accreditation councils.^1^ Traditionally, such assessments have been implicit, unstandardised and based on global, subjective judgements^2^; however, today a variety of direct observation tools (DOTs) are available to guide, delineate and structure formative and summative assessments of clinical skills.^3^ Similarly, a number of DOTs have also been developed or adapted for use specifically in assessing competency in delivering person-centred care (PCC).^4^ These PCC DOTs differ widely in their operationalisations of this construct^4^ and generally show low concurrent validity,^5^ which is not surprising given that PCC is generally recognised as a complex, multidimensional construct^6^ with disparate terminology,^7^ definitions^8 9^ and conceptual frameworks.^10^,^13^ Nonetheless, few PCC DOTs are transparent about their conceptual underpinnings,^4 10^ which blurs what exactly the tool aims to assess and impedes evaluation of its validity and utility in assessing PCC competency. Moreover, content and construct validity have rarely been examined and, paradoxically, patients have not been involved in their development and evaluation.^4^

**Figure 1:**
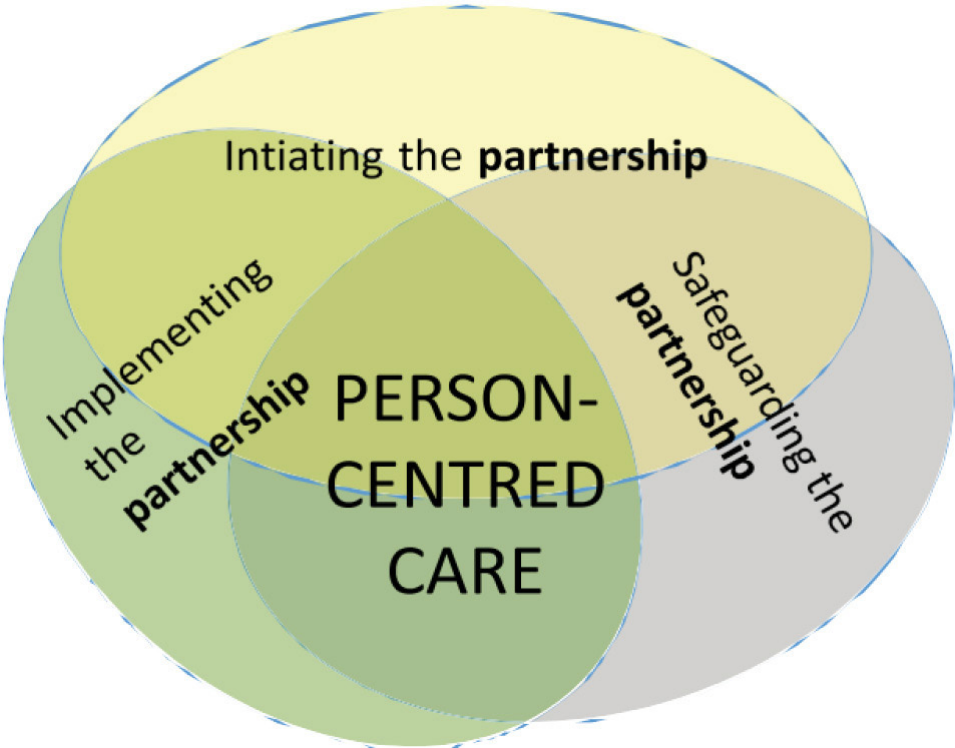
Overview of the Gothenburg Centre for Person-Centred Care (gPCC) framework.^34^ The gPCC framework proposes three major clinical tasks for person-centred care, described as ‘routines’: initiating the partnership, working the partnership and safeguarding the partnership.^14^

Mindful of the shortcomings of current PCC DOTs, we set out to construct a new tool grounded in a recognised, evidence-based PCC conceptual framework and developed with input from both patients and health professionals. The Gothenburg direct observation tool for assessing PCC (GDOT-PCC) is rooted in the Gothenburg University Centre for Person-Centred Care conceptual framework (gPCC; figure 1).^14^ The gPCC has been widely implemented in Sweden, as well as in other parts of Scandinavia, the UK and Poland, and^15 16^ is also the most frequently used framework in research on person-centred interventions^17^ and was influential in the development of the European standard for patient involvement in healthcare.^18^

Recently, we reported that the tool showed satisfactory content validity and usability as assessed by both patients and healthcare professionals.^19^ To be useful for assessing PCC in healthcare education and practice, assessment tools need to demonstrate good inter-rater reliability.^20^ Good inter-rater reliability indicates that a tool is robust to changes in raters, that is, that the tool is less susceptible to measurement error due to variation in rater judgements.^21^

### Aim

To assess the inter-rater reliability of the 15 domains of the GDOT-PCC for assessing healthcare professionals’ competency in delivering PCC.

## Methods

### Instrument

In constructing the GDOT-PCC, the formal goals and tasks of PCC outlined in the gPCC framework (figure 1) were supplemented with insights from interview studies with patients and health professionals regarding how health providers should be and what skills they need to effectively accomplish those goals and tasks in everyday practice.^22 23^ The gPCC proposes three major clinical tasks for PCC, described as ‘routines’: initiating the partnership, working the partnership and safeguarding the partnership.^14^ The first routine seeks to initiate a partnership with the patient by eliciting the patient narrative, that is, the patients’ personal account of their illness, symptoms and their impacts on their daily lives. The second routine seeks to use and further the partnership through, for example, shared decision making, so that providers and patients may effectively cooperate to develop and achieve agreed goals. The third routine aims to safeguard the partnership by ensuring that the narrative is documented regarding the patient’s preferences, values, needs and goals.

The 53-item GDOT-PCC (online supplemental material) assesses 15 PCC domains divided into four major areas: ‘PCC activities’, ‘clinician manner’, ‘clinician skills’ and ‘PCC goals’. The area ‘PCC activities’ includes tasks and goals partitioned in eight domains describing activities to be accomplished in clinician-patient interactions outlined in the three gPCC routines. Three PCC activity domains, personal connection, co-setting agenda and understanding patient perspective, are intended to assess the gPCC routine initiating the partnership by including behaviours needed to set the stage for and elicit the patient narrative. Four PCC activity domains, information needs, shared decision-making, psychosocial needs and personal capabilities, are intended to assess the routine working of the partnership through behaviours seen to capitalise on and strengthen the partnership. The gPCC routine safeguarding the partnership was exemplified by the PCC activity domain co-planning and documentation.

The area ‘clinician manner’ is a single domain and includes nine behavioural indicators, for example, respectful, which the clinician should demonstrate through verbal and/or non-verbal behaviours. The area ‘clinician skills’ comprises the domains perceptual and behavioural skills, for example, speaks in a manner appropriate to the patient’s level of understanding. Neither of these areas is formally represented in the gPCC framework. The area ‘PCC goals’ includes four overall PCC goals to be achieved: patient participation, trust, partnership and shared documentation.

All ratings are made on a bipolar, 5-point rating scale ranging from very unsatisfactory to very satisfactory, labelled −−, −, −/+, +, ++. A response option labelled ‘doesn’t do’ is provided for use with items in the PCC activity area when the activity is not observable but is appropriate within the context of the consultation; this option is not available for the other assessment areas. A field for rater notes is provided beside each domain.

The GDOT-PCC offers several advantages compared with currently available tools: patients were involved in its development and evaluation; it is based on an established and tested PCC framework; it assesses PCC comprehensively; and it is easy to use and requires little or no rater training.

### Design

This was an observational, fully-crossed inter-rater reliability study.

### Participants

Six healthcare professionals, all women, with direct experience of working with PCC in clinical practice were recruited between October 2022 and December 2022 using purposeful sampling and were selected to represent a variety of clinical disciplines. All had experience in teaching and were affiliated with academic institutions. See table 1 for a description of the participants/raters.

**Table 1:** Rater characteristics

Gender	Profession	Length of clinical experience (years)
Female	Registered nurse	45
Female	Registered nurse	30
Female	Registered nurse	11
Female	Physician	5
Female	Physician	17
Female	Psychologist	2

### Data collection procedures

Ratings were performed during the recruitment period. The first author (NE) contacted the participants by email explaining the purpose and procedures of the study. When they had agreed to take part in the study, they received a new email with the instrument, links to 10 video-recorded consultations and instructions explaining how to use the GDOT-PCC.

The videos comprised 10 simulated, 3–15 min consultations selected to cover a range of PCC performance, from poor to good delivery. They were also selected to include different categories of healthcare providers, different purposes for the consultations and conducted in different clinical settings (table 2). The participants all individually rated each filmed interaction and viewed them in a different order to counteract training effects. Participants returned their ratings via email to NE.

**Table 2:** Video-film descriptions

Video-film	Profession	Setting	Purpose of consultation	Length (min)
1	Male physician	Primary care	Male patient has a cold	6.31
2	Female physician	Primary care	Male patient has shoulder pain and anxiety	5.46
3	Female registered nurse	Hospital	Information to a female patient regarding an examination	6.15
4	Female registered nurse	Hospital	Discharge conversation with a female patient	4.02
5	Female physician	Hospital	Transition to end-of-life care with a male patient	9.49
6	Male physician	Hospital	Female patient receives information about a severe illness	15.02
7	Female physician	Hospital	Male patient with depression	5.05
8	Male physician	Hospital	Male patient with psychosis	7.15
9	Male physician	Primary care	Follow-up meeting with a male patient with autism	4.25
10	Male physician	Hospital	Follow-up meeting with a male patient after medicine changes	3.00

### Analysis

Sample size was estimated to 10 interactions using Bonnet’s formula,^24^ with an expected reliability of 0.85, 95% confidence level, precision±0.15 and six raters.

Item ratings were coded such that −− was coded as 1, − as 2, −/+ as 3, + as 4 and ++ as 5. The ‘doesn’t do’ response option, provided for the PCC activities items, was coded as 0. Items in the areas of manner, skills and goals did not have this response option.

The internal consistency of all a priori, multi-item domains was estimated with Cronbach’s α. An α value ≥0.70 was considered to indicate acceptable consistency.^25^ Mean ratings were subsequently calculated for all multi-item domains.

Inter-rater reliability was assessed for each of the 15 domains using two-way mixed, absolute agreement, average-measures intraclass correlations (ICC) with associated CIs,^26^ using mean values for multi-item domains and recoded ratings for single item domains. ICC values were interpreted according to cut-offs proposed by Cicchetti,^27^ where ICC values <0.40 indicate poor agreement, 0.40–0.59 indicate fair agreement, 0.60–0.74 good agreement, and 0.75–1.0 excellent agreement.

All analyses were performed using IBM SPSS Statistics (V.29).

### Patient and public involvement

The study was conducted within the University of GPCC, where patient and public involvement is essential. Patients and patient representatives were involved in the development and evaluation of the GDOT-PCC.

### Ethical considerations

Ethical approval was received from the Swedish Ethical Review Authority for this study (Dnr: 1004-17, T2022-05766-02). The participants gave their written consent, were informed that their participation was voluntary and that they had the right to withdraw at any time without giving reasons.

## Results

The domains planning and documentation and documentation were excluded from analyses due to insufficient evaluable data. Cronbach’s α was acceptable (>0.70) for all remaining multi-item domains. ICC values indicated excellent inter-rater agreement (ICC ≥0.75) for 11 of the 13 evaluated domains (table 3). However, CIs around the point estimates were generally wide and the lower bounds lay within the good range (ICC=0.60–0.74) for six domains and fair (ICC=0.40–0.59) for the other six, suggesting some caution in interpreting the point estimates alone. The ICC for the domain patient perspective was non-informative due to its particularly wide CIs (ICC=0.74 (0.39–0.92)).

**Table 3:** Inter-rater reliability

Areas	Domains	#items	Cronbach’s α	ICC (95% CI)*
Activities	Personal connection	3	0.75	0.81 (0.55–0.94)
	Co-setting agenda	3	0.91	0.93 (0.82–0.98)
	Understanding patient perspective	8	0.95	0.74 (0.39–0.92)
	Information needs	3	0.86	0.88 (0.74–0.96)
	Shared decision-making	4	0.91	0.83 (0.60–0.95)
	Psychosocial needs	2	0.78	0.81 (0.55–0.94)
	Personal capabilities	4	0.89	0.82 (0.59–0.95)
	Co-planning and documentation	7	–	–
Manner	Manner	9	0.93	0.89 (0.74–0.97)
Skills	Perceptual	1		0.86 (0.67–0.96)
	Behavioural	4	0.95	0.78 (0.49–0.93)
Goals	Activation	1		0.80 (0.53–0.94)
	Trust	2	0.93	0.83 (0.60–0.90)
	Partnership	1		0.82 (0.52–0.95)
	Shared documentation	1		–

* Absolute agreement.

## Discussion

The present study examined the internal consistency and inter-rater reliability of the 15 a priori domains comprising a revised, observation-based tool for assessing healthcare professionals’ competence in delivering PCC. Our analyses indicated acceptable internal consistency for all evaluable multi-item domains and good-to-excellent rater agreement on six of the domains and fair-to-excellent agreement on six others. Reliability estimates were non-informative for the patient perspective domain by virtue of its wide CI. In general, the magnitudes of our reliability estimates were comparable with or exceeded those reported for other PCC DOTs.^4^

Although reliability is often conceived as a characteristic inherent to an assessment tool, reliability estimates may be influenced by a number of methodological factors unrelated to the observation tool per se, such as sample homogeneity, unit of analysis,^26^ extent of rater training,^10^ raters’ professional background^28^ and other rater characteristics/idiosyncrasies.^1^ For example, rater training is known to improve inter-rater reliability.^29^ In this study, the raters received no training whatsoever, and it is conceivable that had training been provided, inter-rater reliability would have improved. On the other hand, the relatively good reliability demonstrated for most of the domains suggests that the tool may be used with little or no rater training, which may be advantageous in applications where cost and time constraints prohibit the use of established tools requiring long and intensive training, such as the Roter interaction analysis system.^30^

Inter-rater reliability naturally also depends on factors intrinsic to the instrument. For example, factors such as ambiguities in concept definitions, poorly defined target behaviours or including behaviours not readily observable by raters may negatively affect reliability^31^,^33^ and may also explain lower reliabilities associated with some domains in our tool, particularly the domain patient perspective.

The GDOT-PCC was developed primarily for formative use in healthcare education, training and quality improvement applications to guide and structure learning by providing benchmarks to orient and give feedback to reinforce learners. For more high-stakes applications, the tool will need to undergo more rigorous reliability and validity evaluations. Although designed for general use, the tool will also need to be evaluated in different clinical settings, contexts and disciplines.

### Strengths and limitations

A strength of the study is that sample size requirements were estimated using a recognised method designed specifically for use in ICC studies and which allows for trade-offs between number of raters and subjects. Determining sample size is a critical consideration in the design of any study using inferential statistics, yet few studies assessing inter-rater reliability of PCC DOTs have reported sample size determination.^4^ As sample size requirements decrease with increasing numbers of raters,^27^ we decided it would be more feasible to include a larger number of raters than to include a large number of video-recorded interactions to achieve adequate power. This decision likely impacted the widths of our CI around the point estimates as CI normally decrease with increasing sample size.

There are several limitations to this study. The participants were all women, and so our results may not be generalisable to men; however, they represented a number of professions and clinical experience levels. No subanalyses were performed to determine if professional background or length of clinical experience were associated with inter-rater agreement.

Another limitation is that the sampled consultations included too few examples of documentation behaviours to be able to assess inter-rater reliabilities associated with the domains planning and documentation and documentation. Moreover, ratings were performed using video-recorded consultations; hence, our results may not apply to real-time assessments.

## Conclusion

Most of our inter-rater reliability estimates were high and comparable to or exceeded those of existing observation-based tools for assessing PCC, indicating their reliability for use in education and quality improvement. However, reliabilities for the two documentation domains, as well as the patient perspective domain, need to be reassessed in larger, better-designed studies.

## Supplementary material

10.1136/bmjopen-2024-096576 online supplemental file 1

## Data Availability

All data relevant to the study are included in the article or uploaded as supplementary information.
