# Supplementary material for: Gothenburg direct observation tool for assessing person-centred care (GDOT-PCC): evaluation of inter-rater reliability
Source: BMJ Open. 2025 Apr 17;15(4):e096576. doi: 10.1136/bmjopen-2024-096576 (PMC12007047; doi:10.1136/bmjopen-2024-096576)
Supplement: online supplemental file 1 [file bmjopen-15-4-s001.docx]

| **Activities** | | **Actions** | **Doesn’t do** | **- -** | **-** | **-/+** | **+** | **++** | **Comment** |
| --- | --- | --- | --- | --- | --- | --- | --- | --- | --- |
| Makes a personal connection | | Greets patient in a warm and respectful manner |  |  |  |  |  |  |  |
|  |  | Attempts to make patient feel at ease |  |  |  |  |  |  |  |
|  |  | Is responsive to patient’s emotional and physical state |  |  |  |  |  |  |  |
|  |  | | | | | | | | |
| Co-sets agenda | | Seeks to elicit all patient’s aims and reasons for meeting |  |  |  |  |  |  |  |
|  |  | Clinician explains their agenda for today's meeting |  |  |  |  |  |  |  |
|  |  | Discusses and prioritizes agenda items. |  |  |  |  |  |  |  |
|  |  | | | | | | | | |
| Seeks to understand patient’s perspective | | Actively and attentively listens to patient’s concerns |  |  |  |  |  |  |  |
|  |  | “ feelings |  |  |  |  |  |  |  |
|  |  | “ illness beliefs |  |  |  |  |  |  |  |
|  |  | “ illness experiences |  |  |  |  |  |  |  |
|  |  | “ psychosocial impacts of illness |  |  |  |  |  |  |  |
|  |  | “ treatment goals |  |  |  |  |  |  |  |
|  |  | Verifies that he/she understands patient’s viewpoints correctly |  |  |  |  |  |  |  |
|  |  | Validates patient’s perspective |  |  |  |  |  |  |  |
|  |  | | | | | | | | |
| Attends to patient’s information needs | | Gives information appropriate and relevant to the patient’s needs and wants |  |  |  |  |  |  |  |
|  |  | Gives information in a manner that the patient can understand |  |  |  |  |  |  |  |
|  |  | Clarifies own reasoning and uncertainty |  |  |  |  |  |  |  |
|  |  | | | | | | | | |
| Finds common ground/ engages in shared decision-making | | Identifies and synthesizes areas of agreement and disagreement |  |  |  |  |  |  |  |
|  |  | Seeks to reconcile and align patient-clinician understanding and goals |  |  |  |  |  |  |  |
|  |  | Proposes and discusses care and treatment options compatible with patient’s wants |  |  |  |  |  |  |  |
|  |  | Solicits patient’s suggestions, opinions, preferences |  |  |  |  |  |  |  |
|  |  | | | | | | | | |
| Attends to patient’s psychosocial needs | | Identifies and discusses emotional and social impacts and support needs |  |  |  |  |  |  |  |
|  | | Checks how patient perceives diagnosis and treatment information |  |  |  |  |  |  |  |
|  |  | | | | | | | | |
| Identifies and supports patient’s personal capabilities | | Listens actively to be able to identify resources and capabilities together |  |  |  |  |  |  |  |
|  | | Checks to see that the patient´s resources/capabilities have been identified |  |  |  |  |  |  |  |
|  | | Helps patient discover personal capabilities/resources for coping with illness |  |  |  |  |  |  |  |
|  |  | Discusses how capabilities and resources may be utilized |  |  |  |  |  |  |  |
|  |  | | | | | | | | |
| Co-plans and documents care | | Co-plans and co-documents steps to achieve and monitor shared goals |  |  |  |  |  |  |  |
|  |  | Solicits and encourages patient’s contribution |  |  |  |  |  |  |  |
|  |  | Discusses roles and division of responsibilities |  |  |  |  |  |  |  |
|  |  | Writes journal entries in language patient understands |  |  |  |  |  |  |  |
|  |  | Checks if patient perspectives are fully documented |  |  |  |  |  |  |  |
|  |  | Seeks final approval of documentation |  |  |  |  |  |  |  |
|  |  | Ensures patient access to documentation |  |  |  |  |  |  |  |

| **Manner** | | | **Example behaviors** | **- -** | **-** | **-/+** | **+** | **++** | **Comments** |
| --- | --- | --- | --- | --- | --- | --- | --- | --- | --- |
| Courteous | | | *Verbal*: Introduces him/herself; addresses patient by name  *Nonverbal*: Makes eye contact; knocks before entering room |  |  |  |  |  |  |
|  |  |  | | | | | | | |
| Attentive and interested | | | *Verbal*: Shows interest in patient by e.g., “I’m curious about…”, “Please tell me more about…”  *Nonverbal*: Maintains appropriate eye contact; forward lean; open posture; body oriented toward patient |  |  |  |  |  |  |
|  |  |  | | | | | | | |
| Unhurried | | | *Verbal*: Engages in small talk  *Nonverbal:* Sits; respects pauses in conversation |  |  |  |  |  |  |
|  |  |  | | | | | | | |
| Caring/ empathic | | | *Verbal*: Responds to emotional cues e.g., ”That must be … frightening, disturbing, painful…”; shows concern for patient’s physical and mental comfort, e.g., “Are you comfortable in that chair”  *Nonverbal*: Touch; smile; nods*,* soft voice tone |  |  |  |  |  |  |
|  |  |  | | | | | | | |
| Encouraging | | | *Verbal*: Uses open-ended questions; uses phrases, e.g., “Please tell me (more) about…”, “Go on”  *Nonverbal*: nods; gestures |  |  |  |  |  |  |
|  |  |  | | | | | | | |
| Respectful | | | *Verbal*: Is non-judgmental (suspends judgment), positive and affirming  *Nonverbal*: Doesn´t interrupt; shows turn-taking |  |  |  |  |  |  |
|  |  |  | | | | | | | |
| Genuine | | | *Nonverbal:*  Uses voice tone, gestures and facial expressions congruent with verbal message |  |  |  |  |  |  |
|  |  |  | | | | | | | |
| Altruistic/ Committed | | | *Verbal:* shows willingness to do more than required, shows no self-interest |  |  |  |  |  |  |
|  |  |  | | | | | | | |
| Forthright/ honest | | | *Verbal*: Admits uncertainty; explains own negative behavior, e.g. why stressed, irritated, etc |  |  |  |  |  |  |

| **Skills** | | | **Description** | **- -** | **-** | **-/+** | **+** | **++** | **Comments** |
| --- | --- | --- | --- | --- | --- | --- | --- | --- | --- |
| Perceptual | | | Carefully monitors and interprets patient’s nonverbal emotional cues |  |  |  |  |  |  |
|  |  |  | | | | | | | |
| Behavioral | | | Speaks in a manner appropriate to the patient’s level of understanding |  |  |  |  |  |  |
|  |  |  | Avoids jargon |  |  |  |  |  |  |
|  |  |  | Uses active, reflective and empathic listening skills |  |  |  |  |  |  |
|  |  |  | Effectively paraphrases and summarizes |  |  |  |  |  |  |

| **Outcomes** | | | **Indicators** | **- -** | **-** | **-/+** | **+** | **++** | **Comments** |
| --- | --- | --- | --- | --- | --- | --- | --- | --- | --- |
| Patient activation | | | Gives opportunities and encourages patient to talk (clinician doesn’t dominate conversation) |  |  |  |  |  |  |
|  |  |  | | | | | | | |
| Patient trust | | | Free flow of conversation (turn-taking) |  |  |  |  |  |  |
|  |  |  | Patient freely and actively voices concerns, expectations, beliefs, opinions, suggestions, preferences |  |  |  |  |  |  |
|  |  |  | Transparent documentation |  |  |  |  |  |  |
|  | | |  |  |  |  |  |  |  |
| Partnership | | | The patient feels like an active partner |  |  |  |  |  |  |
|  | | |  |  |  |  |  |  |  |
| Shared documentation | | | Transparent documentation |  |  |  |  |  |  |
