# Supplementary material for: Gothenburg direct observation tool for assessing person-centred care (GDOT-PCC): evaluation of inter-rater reliability
Source: BMJ Open. 2025 Apr 17;15(4):e096576. doi: 10.1136/bmjopen-2024-096576 (PMC12007047; doi:10.1136/bmjopen-2024-096576)
Supplement: Supplementary file 2 [file bmjopen-2024-096576.reviewer_comments.pdf]

PEER REVIEW HISTORY

BMJ Open publishes all reviews undertaken for accepted manuscripts. Reviewers are asked to complete a checklist review form and are provided with free text boxes to elaborate on their assessment. These free text comments are reproduced below.

ARTICLE DETAILS

Title (Provisional)

The Gothenburg Direct Observation Tool for assessing Person-Centred Care (GDOT-PCC): evaluation of inter-rater reliability

Authors

Ekman, Nina; Fors, Andreas; Moons, Philip; Taft, Charles

VERSION 1 - REVIEW

**Reviewer**	**1**
**Name**	**Burgers, JS**
**Affiliation**	**Nederlands Huisartsen Genootschap**
**Date**	**16-Jan-2025**
**COI**	**None**

This study evaluates the internal consistency and inter-rater reliability of a tool for assessing person-centred care principles in general practice. The findings of the study on the tool's content and usability have been published in BMJ Open 2024. The tool can be used in the context of training and education.

Major comments

1. The focus of this paper is limited as it only studied two features of an instrument as part of its validity and reliability. Therefore, it is not possible to assess the tool in context. Moreover, the tool itself has not been included in the submitted materials. The description in the paper does not cover the items, leaving the reader in the dark about the specific content of the tool.

2. The selection of the 10 videos and the characteristics of the patients and health providers in these videos are not described. They should reflect the consultations in daily general practice but this cannot be assessed.

Specific comments

Abstract

Methods: be more concrete about the tool, e.g. including the names of the four areas

Results: the range of CIs should be included in the Results. Formulate the findings as neutral as possible. Include the name of the two domains with poor inter-rater agreement.

Discussion: descriptive facts of the tool (e.g., based on an established framework) should be included in the methods.

Main text

Introduction: 'obfuscate' is difficult to understand. Replace it by another term.

Methods – instrument: the text is the same as in the paper previously published in BMJ Open. be clear that the 'doesn't do' option applies to the 'PCC activities' area.

Methods - design: this should be the first paragraph of the Methods. 'Fully crossed' is an unusual term for a design. It is an observational study of videos recording consultations in general practice.

Methods – participants: did they have experience in teaching or affiliated with academic institutions?

Methods – data collection: explain how you selected the 10 videos and the patient and GP characteristics involved. Information about the duration of each video and the topic of the consultations is also relevant. Information about the video could be presented in a table. Explain why you also selected a video of only 3 minutes. What was the topic of that consultation? Do these consultations represent daily general practice? Please also consider whether the patient knows the GP and vice versa and for how long. This could be addressed in the Discussion.

Methods – PPI: please specify how patients were selected and what and how they contributed.

Results: a table with descriptive scoring data on the items and domains could be added. It would be informative to know to what extent person-centred care was delivered in the 10 consultations and whether the scores vary between items and domains.

Discussion: All participants were women, but most (teaching) HCPs in Sweden may be female. Information about the gender balance in practice is lacking. Implications for education, practice, and policy should be added.

Conclusion: descriptive facts of the tool (e.g., based on an established framework) should be included in the methods. ‘It assesses PCC holistically’ is bad English. The term ‘comprehensive’ could be used.

**Reviewer**	**2**
**Name**	**Matthews, Elizabeth**
**Affiliation**	**Fordham University**
**Date**	**05-Feb-2025**
**COI**	**None**

Thank you for the opportunity to review this manuscript. Defining and measuring person-centered care remains a challenge in the field, and this research therefore offers a significant contribution to the literature. Overall the manuscript is clear, well written and rigorous. The inclusion of service users in the development of this measure is a particular strength, as is its foundation in an established conceptual framework for PCC. I have several minor to moderate comments:

1. As described above, rooting the scale in an established conceptual framework for PCC is a strength of this study. A table or figure that illustrates the domains of the University of Gothenburg Centre for Person-Centred Care (GPCC) conceptual framework and maps scale items (or a sample of items) onto these dimension would be very helpful. At a minimum, a brief description of the foundational PCC framework would help orient readers who are otherwise familiar.

2. More information about how the measure is scored would be helpful. Relatedly, I am interested in the ‘doesn’t do’ option, which indicates that a behavior was not observed. It seems that this could have two different implications for measuring PCC. First, is it possible that behaviors were not observed because they were not clinically indicated (eg: shared decision making might not need to occur at all visits)? This does not necessarily indicate the absence of PCC. Other components of PCC may always be warranted (eg: characteristics of provider’s manner or listening style), so the absence of these behaviors might signal that providers are not being person-centered. I am curious how you approached those two differences, if at all.

3. I am also curious about the videos used to rate PCC. What was the nature and content of these interactions, and how were they chosen? Specifically I am wondering if there was an intentional selection based on their anticipated inclusion (or absence) of PCC behaviors. I

also wonder if the length of the interaction was associated with PCC scores, as fewer behaviors may be observed in a 3 minute clip versus a 15 minute clip.

4. You mention that in addition to ratings, providers could also include notes next to their numeric value. I wonder what information was gleaned from these data in ways that would help contextualize some of your findings about IRR

5. I would also like to see some discussion about the intended application of this tool in healthcare and related contexts. Is this tool designed to be generalizable across settings, contexts and disciplines? (for example, would this be applicable within mental health? Specialty care? Consultations versus routine maintenance visits?)

Thank you again for the opportunity to review this important work.

VERSION 1 - AUTHOR RESPONSE

**Reviewer #1**	**Remarks:**
This study evaluates the internal consistency and inter-rater reliability of a tool for assessing person-centred care principles in general practice. The findings of the study on the tool's content and usability have been published in BMJ Open 2024. The tool can be used in the context of training and education.	Thank you for your very helpful comments.
The focus of this paper is limited as it only studied two features of an instrument as part of its validity and reliability. Therefore, it is not possible to assess the tool in context. Moreover, the tool itself has not been included in the submitted materials. The description in the paper does not cover the items, leaving the reader in the dark about the specific content of the tool.	We have now submitted the tool as supplementary material.
The selection of the 10 videos and the characteristics of the patients and health providers in these videos are not described. They should reflect the consultations in daily general practice but this cannot be assessed.	We have now added a table with the information you request.
**Abstract**<br>Methods: be more concrete about the tool, e.g. including the names of the four areas	We have added more info about the tool, including the names of the 4 areas.

Results: the range of CIs should be included in the Results. Formulate the findings as neutral as possible. Include the name of the two domains with poor inter-rater agreement.	We have added the CI range for the domain with poor agreement, along with its name, as well as the Cicchetti's cutoff ranges for good and fair agreement; however, due to space limitations of the abstract we have not included ICCs and 95% CI for all 15 domains.
Discussion: descriptive facts of the tool (e.g., based on an established framework) should be included in the methods.	This is now in the methods of the main text
**Main text**<br>Introduction: 'obfuscate' is difficult to understand. Replace it by another term.	We have replaced it with "blurred".
Methods – instrument: the text is the same as in the paper previously published in BMJ Open. be clear that the 'doesn't do' option applies to the 'PCC activities' area.	Thanks for making us aware of this. The text has been corrected and revised. We have clarified that this scoring option applies only to PCC activities.
Methods - design: this should be the first paragraph of the Methods. 'Fully crossed' is an unusual term for a design. It is an observational study of videos recording consultations in general practice.	We have now moved the design to the first paragraph of the Methods. We have now described the study as an observational, fully-crossed inter-rater reliability study. We understand that fully-crossed is an unusual term for a design but we respectfully prefer to keep it since it is important information and advantageous in IRR studies that all raters rated all interactions against all items.
Methods – participants: did they have experience in teaching or affiliated with academic institutions?	Yes, all the participants had experience in teaching and were affiliated with academic institutions. This information has been added to the text.
Methods – data collection: explain how you selected the 10 videos and the patient and GP characteristics involved. Information about the duration of each video and the topic of the consultations is also relevant. Information about the video could be presented in a table. Explain why you also selected a video of only 3 minutes. What was the topic of that consultation? Do these consultations represent daily general practice? Please also consider whether the patient knows	Thank you for raising this issue. We have now added a table describing the videos and explained the selection criteria in the text.

the GP and vice versa and for how long. This could be addressed in the Discussion.	
Methods – PPI: please specify how patients were selected and what and how they contributed.	The consultations were simulated and no patients were involved in the study. On the other hand, patients participated in developing and testing the tool.
Results: a table with descriptive scoring data on the items and domains could be added. It would be informative to know to what extent person-centred care was delivered in the 10 consultations and whether the scores vary between items and domains.	This is a good suggestion; however, since the purpose of this study was to examine the interrater reliability associated with the tool, to be meaningful we would seemingly need to include descriptive statistics for each of the 6 raters for each of the 15 domains for each of the 10 consultations.
Discussion: All participants were women, but most (teaching) HCPs in Sweden may be female. Information about the gender balance in practice is lacking. Implications for education, practice, and policy should be added.	The consultations included roughly equal numbers of male and female health professionals. On the other hand, the participants (raters) may be seen to represent the dominance of females in Swedish healthcare education and quality improvement activities, for which the tool is intended. We mention that the tool may benefit healthcare education initiatives and quality improvement efforts.
Conclusion: descriptive facts of the tool (e.g., based on an established framework) should be included in the methods. 'It assesses PCC holistically' is bad English. The term 'comprehensive' could be used.	This has been moved to the methods<br><br>Holistically has been replaced with comprehensively.
**Reviewer #2:**	**Remarks:**
Thank you for the opportunity to review this manuscript. Defining and measuring person-centered care remains a challenge in the field, and this research therefore offers a significant contribution to the literature. Overall the manuscript is clear, well written and rigorous. The inclusion of service users in the development of this measure is a particular strength, as is its foundation in an established conceptual framework for PCC. I have several minor to moderate comments:	Thank you very much for your comments, they were very helpful!
As described above, rooting the scale in an established conceptual framework for PCC is a	Thanks for pointing this out. We have added a figure and some text that hopefully illustrates the

strength of this study. A table or figure that illustrates the domains of the University of Gothenburg Centre for Person-Centred Care (GPCC) conceptual framework and maps scale items (or a sample of items) onto these dimension would be very helpful. At a minimum, a brief description of the foundational PCC framework would help orient readers who are otherwise familiar.	framework and explains how the domains map onto it.
More information about how the measure is scored would be helpful. Relatedly, I am interested in the ‘doesn’t do’ option, which indicates that a behavior was not observed. It seems that this could have two different implications for measuring PCC. First, is it possible that behaviors were not observed because they were not clinically indicated (eg: shared decision making might not need to occur at all visits)? This does not necessarily indicate the absence of PCC. Other components of PCC may always be warranted (eg: characteristics of provider’s manner or listening style), so the absence of these behaviors might signal that providers are not being person-centered. I am curious how you approached those two differences, if at all.	We are very grateful to you for these comments. We agree that the “doesn’t do” option is potentially problematic for scoring. Our intention was that this option be used only when the behavior was, as you say, clinically indicated, and when not, to not rate the behavior (i.e. missing). To be sure that this was in fact the case we reviewed our data for cases where the “doesn’t do” option was used by some raters, while other raters left them as missing. We also reviewed the video-recorded consultations for evidence to support one or the other rating option. We found that nearly all cases concerned the Co-planning and documentation domain, which comprises co-planning behaviors primarily in conjunction with documenting the visit. As few of the included consultations contained observable documentation activities (e.g. collaborating in medical record writing), it appears that the “doesn’t do” option was consistently not used as intended by two raters re this domain. This was substantiated by raters’ notes in the “comments” section stating that behaviors in the domain were generally “not relevant” for particular consultations. Clearly, we need to improve our instructions to raters. On the other hand, we could not identify any other Activity domain where this problem appeared. We have reanalyzed the data for this domain with “Doesn’t do” recoded as missing for the two raters; however, we do not have enough data to have any real confidence in these results and have removed the Cronbach and ICC values from the results. We have now explained this in the manuscript.

	We found a similar problem with scoring the Goal domain Documentation, where instead of not rating this goal, most raters rated it as very poor (=1). Recoding the ratings left too few evaluable cases for the analyses. This too we have now brought up in the paper (and we will also need to clarify this in rater instructions).<br><br>Re your second comment, we understand you to be asking why we didn't include an option analogous to "doesn't do" for the other domains, as used in the Activity domains. We had originally considered eg Respect to be manifested only as degree on a scale from disrespect to respect. But you make a good point here - the behavioral indicators for Respect may in fact not be performed. We will consider this for a revised version of the tool.
I am also curious about the videos used to rate PCC. What was the nature and content of these interactions, and how were they chosen? Specifically I am wondering if there was an intentional selection based on their anticipated inclusion (or absence) of PCC behaviors. I also wonder if the length of the interaction was associated with PCC scores, as fewer behaviors may be observed in a 3 minute clip versus a 15 minute clip.	We have now presented an overview of the videos in a table. Yes, the videos were selected to cover a range of PCC performance, from poor to good delivery. They were also selected to include different categories of healthcare providers, different purposes for the consultations and in different clinical settings. We have now explained this in the paper.<br><br>Re your second question, although more activities may conceivably be performed during a longer visit (allowing more time for patient participation, shared decision making, information exchange, etc.), a quick inspection of our data suggested that the 6 raters overwhelmingly rated the shortest interaction on par with the longest one on most domains, both of which had the highest PCC ratings on most domains of the 10 videos. This is in line with other studies (better equipped to study the question) showing that eg patient satisfaction is not so much a function of the actual time of the consultation as it is the quality of that time.

You mention that in addition to ratings, providers could also include notes next to their numeric value. I wonder what information was gleaned from these data in ways that would help contextualize some of your findings about IRR	The comment sections were rarely used, possibly because raters were able to view the videos as many times as they chose and therefore didn't need to take notes. Also, we imagine that they would be more useful in real-time observations and in eg education applications where an aim is to give detailed feedback to assesses. However, as mentioned, some raters used this section to explain why they did not rate certain items/ domains – which was invaluable for us in disentangling the use of the doesn't do option.
I would also like to see some discussion about the intended application of this tool in healthcare and related contexts. Is this tool designed to be generalizable across settings, contexts and disciplines? (for example, would this be applicable within mental health? Specialty care? Consultations versus routine maintenance visits?)	The tool was designed for general use. This is one reason we selected videos representing different clinical settings, different disciplines, etc. Naturally, the tool will need to be tested before we can be reasonably confident that it is suitable for general application. We have added a few words about this in the discussion.
Thank you again for the opportunity to review this important work.	Thank you again for your great comments!

VERSION 2 - REVIEW

Reviewer 2

Name Matthews, Elizabeth

Affiliation Fordham University

Date 01-Apr-2025

COI

Thank you for the opportunity to review this revised manuscript. The authors have presented well reasoned and thoughtful responses to the reviewers' comments. The added figure and tables help better contextualize both the scale and its application in this current study. I am glad that my comments were helpful in considering approaches to scoring PCC indicators in presence and absence of these behaviors, and appreciate your detailed response to the questions(s) I posed. This study is a significant contribution to the literature and I have no further feedback or comments.
